# Complicated primary intestinal lymphangiectasia (Waldmann's disease) in a child successfully treated with octreotide: A case report from a low-resource setting

**DOI:** 10.1016/j.amsu.2021.102588

**Published:** 2021-07-29

**Authors:** Zohair El Haddar, Yassine Sbia, Maria Rkain, Noufissa Benajiba, Abdeladim Babakhouya

**Affiliations:** a Department of Pediatrics, Mohammed VI University Hospital, Oujda, Morocco; b Faculty of Medicine and Pharmacy, Mohammed I^st^ University, Oujda, Morocco

**Keywords:** Lymphangiectasia, Octreotide, Waldmann, Enteropathy, Case report

## Abstract

**Introduction and importance:**

The primary intestinal lymphangiectasia is a rare exudative enteropathy of unknown etiology that affects the lymphatic system. It causes lymphedema and malabsorption syndrome by the escape of the lymph and its elements into the intestinal lumen.

**Case presentation:**

A female patient, diagnosed at the age of 11 with Waldmann's disease, has initially manifested chronic diarrhea with a stature-ponderal delay at the age of 6 months old; she was treated for a long time as celiac disease patient. Edematous syndrome, chronic diarrhea, staturo-ponderal delay and asymmetric lymphedema of the upper limb are the main clinical symptoms in this case. In addition, the exclusion of secondary intestinal lymphangiectasia was important for the diagnosis. Before and during her follow-up, the patient presented two complications of the disease: warts and osteomalacia. The patient did not respond to treatment with the low-fat diet; therefore, the need to add treatment with octreotide was necessary, which has given quite pleasant results. Octreotide was the therapeutic choice to treat the patient as she was resistant to the appropriate regimen with clinical improvement; nevertheless, certain biological elements of lymphatic leakage persisted.

**Discussion:**

Waldmann's disease is rare. It can be responsible, besides the typical signs, for complications including warts and osteomalacia. The histopathological study of intestinal biopsies may be normal if lymphangiectasias are localized. The treatment is based on a nutritional diet associated with octreotide. During the patient's follow-up, the evolution after almost two years of the introduction of octreotide compared to the diet alone showed improved outcomes.

**Conclusion:**

The treatment of Waldmann's disease is based on an adapted diet and octreotide. This case highlighted the importance of the long term follow-up in this disease.

## Highlights

- Waldmann's disease is a rare entity of unknown etiopathogenesis that affects the lymphatic system.
- It causes lymphedema and malabsorption syndrome by an escape of the lymph into the intestinal lumen.
- Octreotide associated to an appropriate nutritional regimen improves outcomes.

## 1 Introduction

The Primary intestinal lymphangiectasia, also known as Waldmann's disease, was first described in 1961 by Waldmann [1]. It is a rare entity in pediatrics of unclear etiology to date. It is an exudative enteropathy due to a congenital or obstructive malformation of the intestinal lymphatic drainage system, and secondary the dilation of mucosal and submucosal lymphatic vessels [2]. This causes the leakage of the lymph and its components through the intestinal lumen responsible for clinical and biological symptoms [1]. The presence of paucisymptomatic forms makes its prevalence difficult to define. It is a disease that most often begins at an early age before 3 years old, but several cases have been diagnosed in adolescence or even in adulthood [2]. Edema of the lower limbs and chronic diarrheas are the two main clinical signs, while hypoalbuminemia, hypogammaglobulinemia, and lymphopenia are among the first-line biological signs [3]. This manuscript will shed light on the first Moroccan case with Waldmann's disease, a 13 years old female, who has had complications of osteomalacia and warts. It first appeared as chronic diarrhea; however its evolution was satisfactory with the somatostatin analogue treatment (octreotide).

## 2 Case presentation

The patient is a 13-years-old female from the Rif **rural region of Morocco with a history of a non-consanguineous marriage and without any drug or disease history;** she was hypotrophic and was **exclusively** breastfed for 6 months. Food diversification started after that with boiled vegetables, the rest was introduced starting **at** the age of 2 with a daily intake adapted to her age. The patient has never been operated on and has not received radiotherapy before; she also has no family history of digestive or tumor diseases. She was initially admitted to another hospital, at the age of 6 months, for the management of a staturo-ponderal delay associated with chronic diarrhea that goes back 2 months prior **to** her admission. The appearance was watery, sometimes fatty; the symptoms worsened by the appearance of an abdominal distension following moderate ascites. A CT scan as well as a digestive fibroscopy were done, the diagnosis of celiac disease was retained. The patient was put on a gluten-free diet, stopped after 18 months because of non-improvement and persistent diarrhea. Other clinical symptoms appeared; particularly, lymphoedema of the left upper limb at the age of one year causing the lower limb deformation and a staturo-weight delay to become more pronounced. After 3 years, the patient was lost to follow-up due to financial issues.

**Fig. 1:**
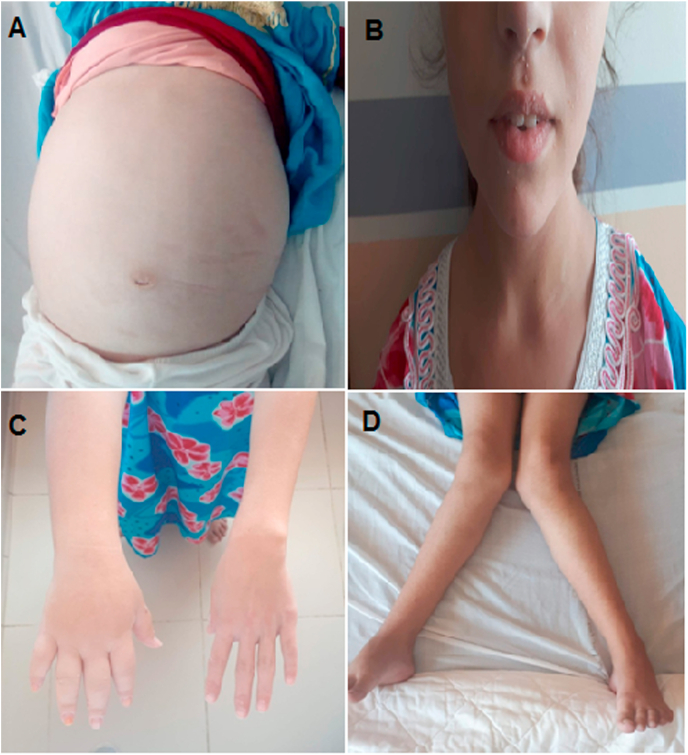
Main clinical manifestations of the patient. (A) abdominal distension, (B) perioral warts, (C) lymphoedema of the upper left limb, and (D) genu valgum.

At the age of 11 years, she was admitted for the first time to our department with oedemato-ascitic syndrome made of very severe abdominal distention, fluid pleural effusion and white soft pitting **edema** of the lower limbs. The clinical examination found: an asthenic girl with no facial dysmorphism or mental retardation, an estimated staturo-ponderal delay of **-4 standard deviation (SD)** for height and −3 SD for weight, an ascites of high abundance with an umbilical perimeter at 100 cm. Lymphedema of the left upper limb with a sausage finger aspect and a dorsal face of the hand rounded into a watch glass was found as well. The patient was apyretic and the urinary strip was negative; the osteoarticular examination found a ***genu valgum*** that dates back to the age of 3 years, with an internal bimalleolar distance of 35 cm as well as a small difference in the length of the two lower limbs. Recurrent warts type molluscum contagiosum in the labial and perioral region and the fingers of both hands **have** also been found (Fig. 1).

The diagnosis of exudative enteropathy such as Waldmann's disease was suspected in the presence of this clinical picture and further investigations were carried out. The malabsorption tests showed microcytic hypochromic anemia with a hemoglobin level of 8.5 g/L, VGM 70 fL TCMH 22 pg, low ferritinemia at 3.02 mg/L, severe hypoalbuminemia at 12g/L, total protein level at 35 g/L, hypocalcemia at 58 mg/L, phosphoremia at 33 mg/L, PTH 1–84 increased to 140.7 pg/ml, vitamin D at 4.8 ng/ml, low cholesterol at 1,05 g/L with HDL at 0.21 g/L and LDL at 0.67 g/L, hypo-gamma-globulinemia at 4.8 g/L, IgG decreased to 2.789 g/L. **The rest of the laboratory work-up was unremarkable.** As a part of the etiological investigations, a serum alpha 1 antitrypsin level of 1.32g/L was found, a faecal alpha 1 antitrypsin level of 0.51g/L with a significantly increased faecal clearance at **224 ml/24H**, which is 10 times the normal.

**Fig. 2:**
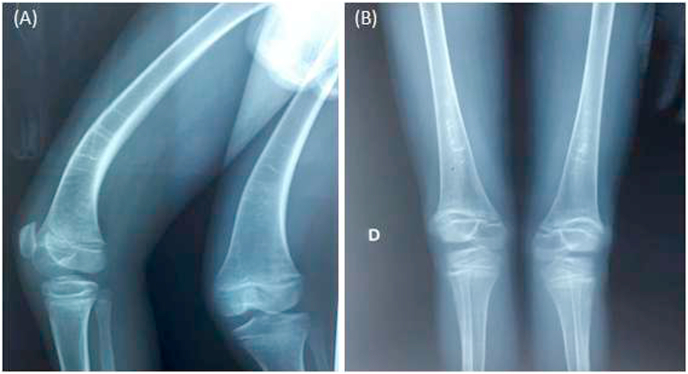
Radiological signs of osteomalacia in our patient (A) (B).

The abdominal ultrasound showed a peritoneal effusion of great abundance with a **thickening of the small bowel's wall.** A thoraco-abdomino-pelvic CT scan showed, in addition to abundant ascites, an inflammatory digestive thickening associated with significant sclerolipomatosis without visible lymphatic obstruction, which is part of inflammatory digestive pathology. The ascites fluid analysis showed milky-looking chylous ascites rich in triglycerides. Fibroscopy revealed congestive gastritis without vilositary atrophy, the histopathological study of duodenal biopsies was normal. A **video-capsule** endoscopy was proposed to the family but due to its shortage in Moroccan hospitals as well as the financial situation of the family, it has not been completed. Other biological and radiological tests were used to rule out differential diagnoses, including *trans*-thoracic ultrasound with no evidence of heart failure, pericarditis or cardiac vein thrombosis, which showed a normal **tuberculosis** assessment, normal stool copro-parasitology and negative HIV and CMV serologies. An x-ray of the lower limbs showed signs of osteomalacia (Fig. 2); an additional teleradiography was requested to study the lower limb deformation. Hence, the diagnosis of the primary intestinal lymphangiectasia (Waldmann's disease) was retained before all clinical and para-clinical signs, and after the elimination of differential diagnoses. The management was difficult at the beginning because of the unavailability of the diet products in Morocco, and the high cost of octreotide for a family who lives in a low-income country. A lot of foreign associations were contacted to help the family provide the treatment from abroad. The patient was initially put on a high-protein and a lifelong low-fat diet with the supplementation of medium-chain triglycerides, with supplements of calcium, iron, vitamin D and A, and some albumin infusions during hypoalbuminemia episodes with a significant oedematous syndrome. However, the evolution after 7 months of follow-up **was** deemed to be a failure; hence, monthly intramuscular injection of slow-release octreotide 20 mg was added, which received good returns with clinical and biological improvement (Table 1, Table 2).

**Table 1:** The clinical evolution in our patient before the diet, before and after octreotide.

Clinical parameter	Before diet	Diet only	Diet + octreotide
Diarrhea	+++	++	–
Lower Extremity Edema	+++	++	–
Umbilical perimeter (cm)	100	93	63
Lymphoedema	+++	+++	+++
Weight	− 3 DS	− 3 DS	− 1 DS
Height	− 4 DS	− 4 DS	- 3DS

+++: important, ++: moderate, -: absent.

**Table 2:** The biological evolution in our patient before the diet, before and after octreotide.

Biological parameter	Before diet	Diet only	Diet + octreotide
Total protein (g/l)	37	35	45
Albumin (g/l)	12	19	28
Lymphocytes (E/μl)	790	490	400
IgG (g/l)	2,7	3,7	3,8
Calcium (mg/l)	58	70	86
Vit D (ng/ml)	4,8	2,6	10,2
Phosphore (mg/l)	38	33	55

An electrocardiography with monitoring of blood pressure and heart rate was performed before and after the introduction of octreotide; the patient didn't show any adverse events. After 2 years of follow-up, **the patient responded and tolerated the treatment well; but with intermittent accessibility to the slow-release octreotide, because it is not available in our country.** Then, during the subsequent follow-up consultations, she did not have pain and subsequent abdominal ultrasounds were proved to be normal. Treatment of lymphedema was symptomatic with multilayer compression bandages. The warts were treated with topical potassium hydroxide aqueous solution and the ***genu valgum*** was treated surgically. The surgical management in our patient was performed by a senior pediatric surgeon with assistance of final year residents.

## 3 Discussion

Waldmann's disease is rare; it is an exudative enteropathy responsible for lymphatic leakage and it is generally diagnosed in childhood, but sometimes in adolescence or adulthood [2]. The diagnosis is based on a set of clinical, biological, radiological, endoscopic and histopathological elements. Clinically, it manifests itself as chronic diarrhea, edematous syndrome by albumin leakage, asymmetric lymphoedema, which most often affects the lower limbs by lymphatic damage, and staturo-ponderal delay [3]. Other symptoms may be present in case of complications, including recurrent or opportunistic infections [4], deficient osteomalacia [5], as well as cutaneous lymphangiectasia [6]. Biologically, as a result of lymphatic leakage, lymphopenia, hypogammaglobulinemia, **hypoproteinemia**, and hypoalbuminemia are almost constant [7], the high clearance of α-1 antitrypsin in the stool is important for the diagnosis [8]. The other biomarkers are mainly a malabsorption syndrome, a decrease in the level of fat-soluble vitamins, hypocalcemia, **hypolipidemia** and an anemia explained by a martial or vitamins B9 or B12 deficiency [7]. In imaging, the thoraco-abdominopelvic CT can eliminate some differential diagnoses; it can **also** show a **nonspecific** thickening of the digestive wall [9]. Fibroscopy can show non-specific signs including congestive intestinal loops, sometimes covered with fibrosis, lymphatics in the form of linear channels on the mucosa leading to the diagnosis of a lymphatic obstacle. Sometimes it shows an accumulation of chylomicrons in the form of whitish villi, and in some cases, fibroscopy can be normal [10]. Video-capsule can be useful for the diagnosis, it can show small yellowish spots like clumps of butter corresponding to lymphangiectasia [11]. The histopathological study of intestinal biopsies may be normal if lymphangiectasias are localized, hence the need to multiply biopsies throughout the small intestine [12]. The aspect that confirms the diagnosis is a dilation of the lymphatic vessels (lymphangiectasias) at the mucosal and sub-mucosal levels [13]. The main complications of Waldmann's disease can be both opportunistic or recurrent infections, as a result of a damage of humoral and cellular immunity by lymphocyte and gammaglobulin leakage [14,15]. Several cases have been reported in the medical literature such as infections with cytomegalovirus, cryptosporidium, salmonella, pneumococcus, cryptococcal meningitis [15,16], that's the case of our patient who has warts probably because of an HPV-like infection.

Secondly, osteomalacia by vitamin D deficiency, calcium, and phosphorus due to the malabsorption of these elements important for bone [17], the symptoms vary from the absence of clinical signs to bone deformities [17]. The patient has a ***genu valgum*** with radiological signs of deficiency, and secondary hyperparathyroidism. Thirdly, non-Hodgkin's lymphoma, which usually appears after 10–15 years of progression [18,19] requires regular monitoring in consultation [20].

The treatment is based on a diet free of long-chain lipids, enriched with protein and medium-chain triglycerides, this diet may be **effective** [21,22]. However, sometimes it doesn't always give good results [23]; thence, the need in some refractory cases to use other treatments such as somatostatin analogue: octreotide. It was first used for the treatment of PIL in 1998 [23], its true mechanism of action remains unknown, the main theories proposed are: The reduction of acetylcholine secretion in the intestinal plexus, affects motility and intestinal absorption [24] as well as causing the Decrease in triglyceride absorption in the thoracic duct [25]. Bac and all proposed as a theory the Inhibition of lymph excretion by the vascular effect of local somatostatin receptors **(SSTR)** [26]. For Klingenberg and all, the mechanism of action is an inhibition of the action of the endocrine and exocrine peptidergic glands of the gastrointestinal tract [27]. The efficacy of treatment with Octreotide has been demonstrated in cases described in medicine literature [23,26,28], some of which have not been found to be more effective than the regimen [29].

During the patient's follow-up, the evolution after almost two years of the introduction of octreotide compared to the diet alone showed a very good evolution of clinical signs. The diarrhea and oedemato-ascitic syndrome disappeared; weight to age improved spectacularly; however, a persistent statural delay as well as lymphedema in the upper body. Biologically, lymphopenia and hypogammaglobulinemia persist; other studies have found the same results, while total protein and albumin levels have increased [26]. In addition to this treatment, other therapeutic options can be used such as parenteral nutrition, anti-plasmin, everolimus and surgery [[30], [31], [32], [33]].

Our patient's family was satisfied with our management. This case was reported based on the latest SCARE guidelines [34].

## 4 Conclusion

Waldmann's disease is a rare condition in pediatrics that must be considered before a picture of exudative enteropathy, endoscopy, and anatomopathological studies can confirm the diagnosis. This condition can be severe by the risk of non-Hodgkin's lymphoma and infectious complications. The main treatment of refractory forms to the appropriate diet is octreotide.

## Ethical approval

Not required for this case report.

## Sources of funding

None.

## Author contribution

Conceptualization: ZEH and AB. Data curation: ZEH and AB. Supervision: AB, NB, and MR. Validation: AB. Writing: ZEH and YS. All authors read and approved the final version of the manuscript.

## Consent

Written informed consent was obtained from the patient’ legal parents for publication of this case report and accompanying images. A copy of the written consent is available for review by the Editor-in-Chief of this journal on request.

## Registration of research studies

Not required for this case report.

## Guarantor

Doctor Zohair El haddar.

## Provenance and peer review

Not commissioned, externally peer-reviewed.

## Declaration of competing interest

The authors report no conflicts of interest
